# A Tensor-Based Structural Damage Identification and Severity Assessment

**DOI:** 10.3390/s18010111

**Published:** 2018-01-02

**Authors:** Ali Anaissi, Mehrisadat Makki Alamdari, Thierry Rakotoarivelo, Nguyen Lu Dang Khoa

**Affiliations:** 1 Faculty of Engineering and IT, The University of Sydney, Sydney, NSW 2006, Australia; ali.anaissi@sydney.edu.au; 2 School of Civil and Environmental Engineering, Universiry of New South Wales, Sydney, NSW 2052, Australia; 3 Data 61, Commonwealth Scientific and Industrial Research Organisation (CSIRO), 13 Garden Street, Eveleigh, NSW 2015, Australia; Thierry.Rakotoarivelo@data61.csiro.au (T.R.); Khoa.Nguyen@data61.csiro.au (N.L.D.K.)

**Keywords:** tensor analysis, damage identification, damage severity assessment, structural health monitoring (SHM)

## Abstract

Early damage detection is critical for a large set of global ageing infrastructure. Structural Health Monitoring systems provide a sensor-based quantitative and objective approach to continuously monitor these structures, as opposed to traditional engineering visual inspection. Analysing these sensed data is one of the major Structural Health Monitoring (SHM) challenges. This paper presents a novel algorithm to detect and assess damage in structures such as bridges. This method applies tensor analysis for data fusion and feature extraction, and further uses one-class support vector machine on this feature to detect anomalies, i.e., structural damage. To evaluate this approach, we collected acceleration data from a sensor-based SHM system, which we deployed on a real bridge and on a laboratory specimen. The results show that our tensor method outperforms a state-of-the-art approach using the wavelet energy spectrum of the measured data. In the specimen case, our approach succeeded in detecting 92.5% of induced damage cases, as opposed to 61.1% for the wavelet-based approach. While our method was applied to bridges, its algorithm and computation can be used on other structures or sensor-data analysis problems, which involve large series of correlated data from multiple sensors.

## 1. Introduction

All civil structures degrade over time, and many also experience harsh environmental and/or excessive operational stress. For most structures such as bridges, the current monitoring practice relies on visual engineering inspections. They use simple tests, which are are expensive, time-consuming, qualitative, often subjective, and only capable of assessing suspicious problems. In the case of bridges, the increase in traffic loading and undetected structural degradation may violate current safety standard requirements. In extreme cases, bridge overloading has led to collapses as in the recent cases of the Lecco overpass in Italy, the Yellow ’Love’ Bridge in Indonesia or the Tolten River in Chile.

Structural Health Monitoring (SHM) systems provide a quantitative, objective, and less expensive alternative to continuously monitor these ageing infrastructures. SHM systems tightly integrate sensor-based data collection, complex data analysis algorithms, and intuitive information presentation software to allow managers and engineers to make informed decisions on a structure’s maintenance and damage mitigation. SHM may provide early damage detection, ongoing condition assessment, and future failure prediction. This contributes to safer structures, less expensive and targeted maintenance tasks, and decreased service interruptions.

Research in SHM is intrinsically interdisciplinary, and spans research areas such as sensor networks, civil engineering, computer science, or data analytics. This paper focuses on one of the major challenges within SHM, namely how to collect and analyse the relevant data to detect and assess existing damage in structures such as bridges.

Thus, this paper presents a novel algorithm based on tensor analysis and one-class learning for structural damage detection. Tensor analysis is used to fuse and extract information collected from multiple sensors instrumented in a structure. These sensors’ measurements usually have a high redundancy and correlation, which two-way matrix analysis may fail to capture all of these correlations and relationships together [1]. In contrast, tensor analysis allows the learning from these highly correlated data in multiple modes at the same time [2]. We build on these contributions and integrate tensor analysis for data fusion with a one class support vector machine (OCSVM) to propose a novel damage detection method.

This work is part of our broader efforts to apply data-driven SHM approaches to real bridges in operation, including the Sydney Harbour Bridge (SHB) [3]. We evaluated the performance of our method using the data collected from one of our SHM deployments on a cable-stayed bridge in operation in Western Sydney, and from one of our laboratory-based experiments on a replica of an SHB substructure. In that latter case, the experimental data were collected using the same sensor system that we deployed on the SHB. This evaluation demonstrated that our novel method provides better damage detection performance than the existing state-of-the-art approach, which is based on wavelet energy spectrum [4].

There are existing reports of SHM system deployments on large-scale bridges. For example, a vibration-based system has been deployed on the Infante D.Henrique Bridge, an arch bridge with a span of 280 m in Portugal. It uses twelve accelerometers to capture the vibration response of the bridge and derives its modal parameters using automated operational modal analysis, which are further used as damage indicative features [5]. In another example, a large-scale SHM system using 2400 low-cost accelerometers and 800 smart sensor nodes has been permanently deployed on the 1.2 km of the Sydney Harbour Bridge in Australia since 2011 [3]. A machine learning algorithm was deployed at the edge of the system on the sensor nodes, and provides successful damage identification [6,7]. Another system of 110 strain gauges has been implemented on the 2160 m Tsing Ma Bridge in Hong Kong [8]. The measured strain data under actual traffic conditions are used to generate the daily stress spectra via a rainflow counting algorithm. The obtained stress spectra is further processed by statistical analysis for identification of fatigue-related damage. As mentioned earlier, our proposed novel method was evaluated using data and/or equipment from such similar real life deployments.

Wireless sensor networks (WSNs) provide a cost effective and logistically less complex deployment alternative than the previous wired-based systems. A recent survey [9] of WSN-based SHM systems identified some examples such as the 70-node system on the Jindo Bridge (South Korea), which uses a threshold approach to damage detection, i.e., an alarm is sent to a gateway node when some data feature exceed a pre-defined threshold [10]. However, WSNs raise other specific challenges, such as energy cost (e.g., battery-powered nodes), wireless channel stability (e.g., data delay, loss, throughput), time synchronisation, or storage and computation constraints. Some solutions were proposed to address some of these issues, such as hybrid wakeup/sleep schemes, cluster-based processing algorithms, or time synchronization error resilient algorithms [9]. While our novel method was evaluated using a wired-based data collection system, it could be readily applicable and deployable to WSN-based systems.

The significant cost of these stationary SHM systems has led researchers to investigate alternatives such as Drive-By-Inspection or Indirect SHM for damage identification on bridges. These approaches use the vibration response of a vehicle passing over the bridge to detect damage. Theoretical and experimental validation of such methods have been described in [11]; however, there is no reported successful real-world deployment to date.

Most recent algorithms proposed to detect and assess damage in structures are based on signal processing and domain expert analysis. Examples of such approaches include the use of the amplitudes of the structure’s natural frequencies [12], wavelet transform-fractality model [13], or subspace methods [14]. As opposed to these recent algorithms, our method is completely data-driven, i.e., it does not rely on domain expert guided signal processing, but rather extract informative features from the data and apply machine learning on them to construct a model for anomaly detection. Thus, it could be potentially applied to a wider range of SHM problems as it does not depend on a specific type of signal (e.g., vibration, strain, acoustic signals).

The remainder of the paper is organized as follows. Section 2 presents the case studies, which provide the data to evaluate our method. Section 3 describes our novel damage identification approach using tensor analysis for data fusion and OCSVM for anomaly detection. Section 4 presents our experimental performance evaluation. Finally, Section 5 concludes this paper with a summary of our contributions.

## 2. Case Studies

### 2.1. The Cable-Stayed Bridge

**Figure 1:**
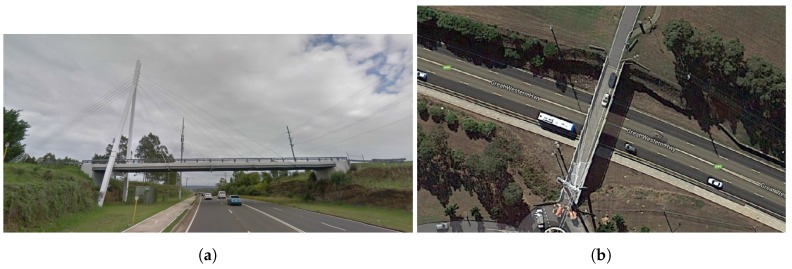
The cable-stayed bridge from our first case study, Western Sydney, Australia (source: ©2017 Google). Side view (**a**) and top view (**b**).

We deployed a sensor-based monitoring system on a cable-stayed bridge in Western Sydney, Australia [15]. This bridge carries one traffic lane and one pedestrian lane. It is 46 m long and connects two university sites across a highway section. It is composed of single deck which is 0.16 m thick and 6.3 m wide. This deck is supported by four I-beam steel girders, and 16 stay cables. These cables are connected to the 33 m mast of the cable-stayed bridge. Figure 1 shows a side and top view of this bridge.

**Figure 2:**
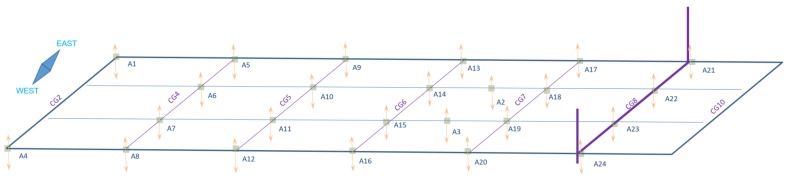
The locations on the bridge’s deck of the 24 Ai accelerometers used in this study. The cross girder *j* of the bridge is displayed as CGj.

Our monitoring system is composed of multiple off-the-shelf sensors, including 29 accelerometers and 28 strain gauges of various types (uniaxial, triaxial, and shear rosette). The locations of these sensors were selected using domain-based knowledge from structural engineers, in order to capture the most relevant response signal from the bridge. In this paper, we are using only features based on accelerations data, and thus we further detail the locations of the accelerometers only. Figure 2 shows the locations of the 24 uniaxial accelerometers (Ai with i∈[1;24]), which were used in this study and installed on the bridge deck. The remaining five accelerometers were installed on four cables and the mast, and are omitted from Figure 2.

These sensors are connected to an HBM Quantum-X data logger (https://www.hbm.com/en/2128/quantumx-compact-universal-data-acquisition-system/) attached to an embedded computer on one side of the bridge. This embedded device provides time synchronization to the data, and stores them temporarily before forwarding via WiFi to a gateway on a nearby building. This gateway then forwards the data over a Virtual Private Network (VPN) to our laboratory. The acceleration data are collected at 600 Hz, with a range of 2 G and a sensitivity of 2 V/G.

This bridge is located on top of a hill (33${}^{\circ }$45${}^{\prime }$50.49${}^{\prime \prime }$ S, 150${}^{\circ }$44${}^{\prime }$31.14${}^{\prime \prime }$ E) and subjected to high wind-induced vibration. The bridge is also located over a busy highway (Great Western HWY), which has a high influence on excitation of the bridge. In this study, we emulated some damage on this bridge as real damage were not available. From a structural engineering perspective, having a large static load at a location of a structure can simulate the reduced stiffness of that location. When measuring the acceleration response of the structure at and around that point, this increased mass produces acceleration measurements that are similar to the ones that would have been produced by damage at that location. Three scenarios have been considered, which includes: no vehicle is placed on the bridge (healthy state), a light vehicle with approximate mass of 3 t is placed on the bridge at different locations (“Car-Damage”) and a bus with approximate mass of 12.5 t is located on the bridge at mid-span (“Bus-Damage”). This emulates a series of several independent damage points, which were used in our evaluation in Section 4.1. The vibration response of the bridge under these scenarios was collected from different times along a day, e.g., the healthy state data are collected at around 2:00 a.m., the car damage data are approximately collected from 11:00 a.m. to 3:00 p.m. and the bus data are collected from 4:00 p.m. to 5:00 p.m. As a result of the time difference between different scenarios, operational and environmental variation of the bridge is highly expected; for instance, just by investigating the closest whether station reports at Penrith, New South Wales [16], a temperature variation of 46%, a humidity variation of 28% and a wind speed variation of 6 km/hr can be observed during the course of measurement. Operational modal analysis using ARTeMIS [17] was conducted on the measured ambient vibration response of the bridge when there was no added mass on the bridge and when a light car was sitting close to the cross girder 5 where maximum of the first bending mode occurred. The fundamental frequency of the structure for these two cases was, respectively, 2.04 Hz and 1.98 Hz, which indicates a drop of only 2.94%. For all of the other cases, where the light car was placed at other locations, the change in the fundamental frequency compared to the healthy case was even smaller than 2.94%, which corresponds to small damage. For the case that the bus was sitting on the deck, the natural frequency of the bridge dropped to 1.80 Hz, which is equivalent to a frequency change of 11.76% compared to the benchmark state.

### 2.2. The Jack Arch Specimen

**Figure 3:**
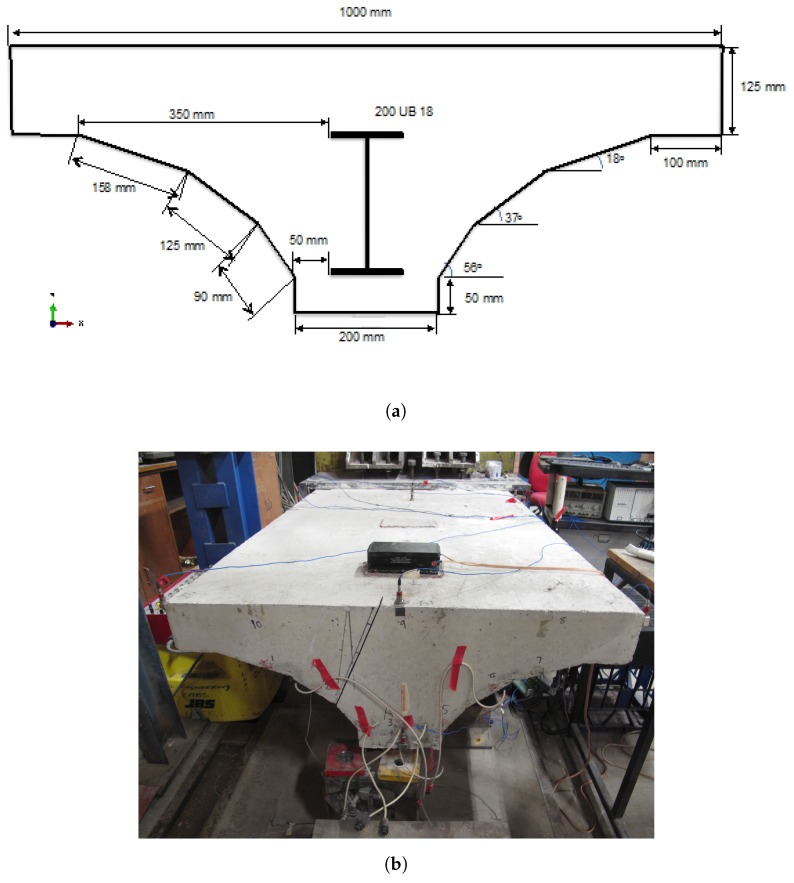
Illustration of the jack arch specimen with attached sensors, (**a**) schematic diagram, (**b**) photo.

For this case study, we built a replica of a structural component (i.e., a jack arch) of the SHB. The real SHB has about 800 of these jack arches located under the bus lane on its eastern side. Our replica specimen was built as a steel reinforced concrete beam with a similar geometry to those on the SHB, and with an I-beam (UB 200-18) embedded inside the concrete as shown in the cross section of Figure 3a. The length of the specimen was 2000 mm, the width was 1000 mm and the depth was 375 mm. The specimen was fixed at one end using a steel bollard to form a cantilever, where 400 mm along the length of the beam were fully clamped. In addition, a support was placed at 1200 mm away from the tip to avoid any cracking occurring in the specimen under self-weight.

The data was collected from two sets of sensor nodes placed on the base of the joint, one node was positioned at the tip while the other was mounted 750 mm away from the tip. There were three accelerometers connected to each sensor node, which were mounted to the left, middle and right sides of the arch, as illustrated in Figure 3b. These sensor nodes and accelerometers are identical to the ones that we deployed on the SHB in another study [3]. The excitation was made using an impact hammer. Once the specimen was triggered by a hammer, the node records data for 3 s at a sampling rate of 500 Hz, resulting in 1500 samples for each event. The variation in the excitation of the structure was taken into account by considering impact loading with different energy levels. This variation experimentally simulated the effect of operational conditions in real world applications.

After testing the benchmark in a healthy condition, a crack was gradually introduced into the specimen with four level of crack dimensions: (75×50) mm${}^{2}$, (150×50) mm${}^{2}$, (225×50) mm${}^{2}$ , and (270×50) mm${}^{2}$. The impact hammer test was conducted again in each damage severity. About 200 events were collected in the healthy condition and in each level of damage severity.

## 3. Method

In this section, we present a method for data fusion and feature extraction using tensor analysis. We then apply an OCSVM algorithm to these tensor-based extracted features in order to detect anomalies in incoming collected data. We also discuss the use of wavelet package energy (WPE) as an alternative state-of-the-art approach for feature extraction. Finally, we present a scheme that connects these described methods to detect and assess the severity of damage on a structure.

**Figure 4:**
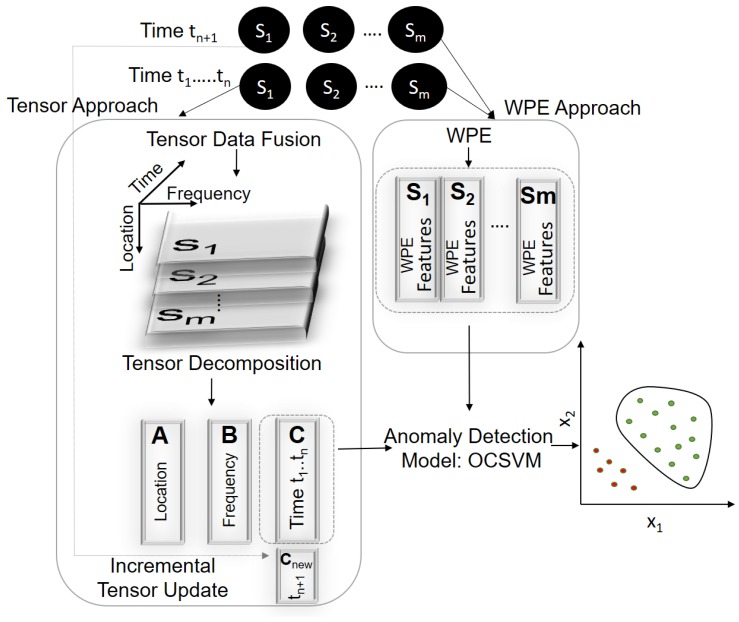
Overview of damage detection using tensor-based approach and an alternate WPE-based approach.

Figure 4 illustrates the steps for both our tensor-based method for damage detection and the WPE-based approach against which we compare our method. On this figure, we have some training data collected during a period ${\left\{t_{i}\right\}}_{i=1}^{n}$ from a healthy structure using *m* sensors denoted by ${\left\{S_{i}\right\}}_{i=1}^{m}$. The tensor approach aggregates these data from *m* sensors in a tensor form and then applies a tensor decomposition technique to extract damage sensitive features represented by the time component. This matrix is used to construct a OCSVM model, which is later used for anomaly detection. When new data from multiple sensors arrive at time $t_{n+1}$, the incremental tensor update step transforms them into an equivalent tensor-based time component, which is then presented to the OCSVM model for damage detection.

The WPE approach, on the other hand, applies a WPE algorithm on each sensor $S={\left\{S_{i}\right\}}_{i=1}^{m}$ to extract the WPE-features. These features are then concatenated into a one feature vector, which is used to construct a different OCSVM model. Section 4 discusses the performance of this alternate model against the one generated by our tensor approach. The following sections provide details of each step in this framework together with the tools and algorithms we used.

### 3.1. Feature Extraction: Tensor Approach

#### 3.1.1. Tensor Data Fusion

**Figure 5:**
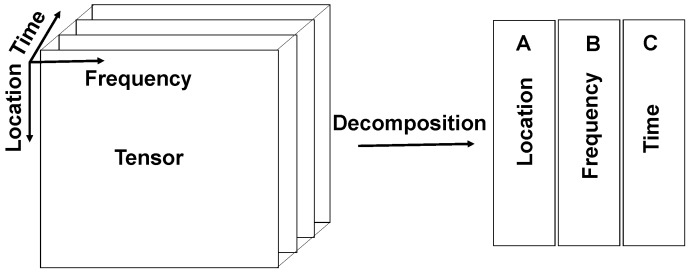
Tensor data with three modes in SHM applications.

In SHM, data are usually collected from a large number of sensors, especially for large civil structures like a long span bridge or a high-rise building. For instance, several accelerometers may be put along a bridge’s spans to measure vibration signals excited by traffic loadings over long periods of time. One traffic event at a specific time produces multiple signals measured by different sensors. These SHM data can be considered as a three-way tensor, i.e., a three dimensional array of (location×frequency×time) as described in Figure 5. However, it is also possible to generalize all the theories for a *n*-way tensor. The frequency in Figure 5 is the measured data in frequency domain (or other types of information extracted from raw measured data). Location represents sensors, and time is data snapshots at different timestamps. Each cell of the tensor is a frequency value extracted from a particular sensor at a certain time. Each slice along the time axis shown in Figure 5 is a frontal slice representing all frequency values across all locations at a particular time.

These measured data from individual sensors are not only correlated with each other in time but also autocorrelated over time. Two-way matrix analysis, as usually used in SHM, can not capture all of these correlation and relationships together [1]. It usually involves a matricization of a multi-way tensor followed by the use of techniques such as principal component analysis (PCA) or singular value decomposition (SVD) to further analyze the data. For example, we can concatenate the frequency data from multiple sensors at a certain time to form a single data instance at that time for anomaly detection in time dimension. However, unfolding the multi-way data and analyzing them using two-way methods may result in information loss and misinterpretation since it breaks the modular structure inherent in the tensor data [1]. In contrast, tensor analysis allows for the learning from these highly correlated data in multiple modes at the same time [2]. It has contributed to successes in many domain applications such as social network and brain data analysis, web mining and information retrieval, or health care analytics [18].

In this work, tensor analysis is used to fuse and extract information from different sensors for damage detection and severity assessment in SHM.

#### 3.1.2. Tensor Decomposition

Tensor decomposition is used to extract latent information in each dimension from tensor data. Two typical approaches used for tensor decomposition are CP decomposition (CANDECOMP/PARAFAC decomposition) and Tucker decomposition [2]. This work adopts the CP method for tensor decomposition due to its ease of interpretation compared with the Tucker method [1].

In case of a three-way tensor $X\in {ℜ}^{I\times J\times K}$, three different matrices are obtained once X is decomposed using CP. Each matrix represents latent information for each mode or dimension. In the case of SHM data as in Figure 5, they are associated with location (denoted matrix *A*), frequency (matrix *B*) and time modes (matrix *C*), respectively. Then, a three-way tensor X is expressed as

(1) $$\begin{array}{c} X\approx \sum_{r=1}^{R}\lambda_{r}\;A_{r}\circ B_{r}\circ C_{r}\equiv \left[\lambda ;A,B,C\right], \end{array}$$

where *R* is the number of latent factors, $A_{r},B_{r}$ and $C_{r}$ are r-th columns of component matrices $A\in {ℜ}^{I\times R}$, $B\in {ℜ}^{J\times R}$ and $C\in {ℜ}^{K\times R}$, and λ is the weight vector so that the columns of A,B,C are normalized to length one. The symbol “∘” represents a vector outer product.

The main purpose of CP decomposition is to minimize the sum of squares of the difference between the tensor X and the model:

(2) $$\begin{array}{c} \min_{A,B,C}∥X-\sum_{r=1}^{R}\lambda_{r}\;A_{r}\circ B_{r}\circ C_{r}{∥}_{f}^{2}, \end{array}$$

where ${∥X∥}_{f}^{2}$ is the norm value, which is the sum squares of all elements of X, and the subscript *f* denotes the Frobenius norm.

The problem defined in Equation (2) is non-convex since it aims to minimize three factor matrices at the same time. However, if we fix two of the matrices, then the problem reduces to a linear least squares problem for solving the third one. Following this approach, the CP decomposition is carried out using an alternating least square (ALS) technique. It iteratively solves each factor matrix by fixing other two matrices using a least square technique until it meets a convergence criterion [2]. The ALS technique is described in Algorithm 1 [2].
**Algorithm 1** CP Decomposition Using Alternating Least Squares**Input**: Tensor $X\in {ℜ}^{I\times J\times K}$, latent factors *R***Output**: Matrices $A\in {ℜ}^{I\times R}$, $B\in {ℜ}^{J\times R}$, $C\in {ℜ}^{K\times R}$, and λ 1: Initialize A,B,C 2: Repeat   3: $A=\underset{A}{\arg\min}\frac{1}{2}{∥X_{\left(1\right)}-A{\left(C⊙B\right)}^{T}∥}^{2}$   4: $B=\underset{B}{\arg\min}\frac{1}{2}{∥X_{\left(2\right)}-B{\left(C⊙A\right)}^{T}∥}^{2}$   5: $C=\underset{C}{\arg\min}\frac{1}{2}{∥X_{\left(3\right)}-C{\left(B⊙A\right)}^{T}∥}^{2}$    (⊙ is the Khatri-Rao product and $X_{\left(i\right)}$ is an unfolding matrix of X in mode *i*) 6: Until convergence criterion is met

Once the convergence criteria is met, the ALS algorithm returns the three matrices *A*, *B* and *C* . As mentioned before, the matrix $C\in {ℜ}^{K\times R}$, which is associated with the time mode, will be used later for constructing an anomaly detection model. This matrix has *K* rows, each of which represents a data instance aggregated from all the sensors at a specific time. This shows how tensor decomposition can be used for data fusion and feature extraction from multiple sensors.

#### 3.1.3. Incremental Tensor Update

When new data arrive (e.g., a frontal slice in time mode), we need to incrementally update the tensor component matrices. For damage detection and assessment, time matrix *C* is utilized. As a result of a new slice in time mode (a matrix of location×frequency), a new row $C_{new}$ will be added to *C*. This paper follows a method proposed by [19] to estimate the $C_{new}$ by fixing two components *A* and *B* as follows:

$$\begin{array}{cc} C & =\arg\min_{C}\frac{1}{2}{∥X_{\left(3\right)}-C{\left(B⊙A\right)}^{T}∥}^{2}\\ & =\arg\min_{C}\frac{1}{2}{∥[\begin{array}{c} X_{old(3)}-C_{old}{\left(B⊙A\right)}^{T}\\ X_{new(3)}-C_{new}{\left(B⊙A\right)}^{T} \end{array}]∥}^{2} \end{array}$$

The new row in time mode $C_{new}$ can be estimated by using only information from newly arrived data $X_{new(3)}$ and matrices *A* and *B* obtained in the training phase:

(3) $$\begin{array}{c} C=[\begin{array}{c} C_{old}\\ C_{new} \end{array}]=[\begin{array}{c} C_{old}\\ X_{new(3)}{\left({\left(B⊙A\right)}^{T}\right)}^{†} \end{array}], \end{array}$$

where † is the matrix pseudo-inverse.

### 3.2. Feature Extraction: Wavelet Packet Energy

To demonstrate the robustness and reliability of our new tensor-based feature for SHM applications, we compare its performance to an alternative approach based on a state-of-the-art feature [4]. One example of such a proven feature for SHM is one using wavelet energy spectrum, which is obtained via wavelet packet decomposition of the original data. This feature has been demonstrated to be sensitive and robust for damage detection at an early stage of development [20].

Wavelet packet decomposition uses a set of low-pass and high-pass filters to decompose a signal into different multi-layers frequency sub-bands, which are mutually independent. It improves the frequency localised capacity and resolution of time domain analysis compared to conventional multi-resolution wavelet analysis. As a result of damage occurrence, the information of each frequency band of the signal decomposed by wavelet packet changes, e.g., the energy of signal in some frequency bands increases while it is reduced in other frequency bands. Therefore, the energy spectrum of the signal in each frequency band contains useful information, which is adopted as a damage sensitive feature.

In this study, first, the wavelet packet decomposition of the signal is conducted in MATLAB using Daubechies 2 wavelet (db2) as mother wavelet with decomposition level of j=4. At level 4, a total of 16 frequency sub-bands will be constructed. The relative energy of each frequency sub-band, e.g., *i* is obtained by normalising the energy of the signal in that frequency sub-band $E_{j}^{i}$ with respect to the total energy, $E_{f},$ as

(4) $$\begin{array}{c} E_{i}=\frac{E_{j}^{i}}{E_{f}}. \end{array}$$

The obtained relative energy at each frequency band is then stored in sequence to construct a vector for a particular sensor,

(5) $$\begin{array}{c} E=(E_{1},E_{2},\ldots ,E_{16}). \end{array}$$

Since, in this study, the response of the structure is measured from multiple sensors, the same exercise is repeated for each sensor and the obtained vectors are concatenated to establish the single feature vector. In Section 4, the performance of this feature is compared with the tensor-based feature.

### 3.3. Anomaly Detection Model: One-Class Support Vector Machine

one class support vector machine (OCSVM) [21] is an extension of the support vector algorithm to the case of unsupervised learning when you only have data from one class. This case represents the main challenge in our application where only data instances forming one state i.e., healthy state are available, and the samples from other classes are very few or do not exist. In this sense, OCSVM is well suited to this kind of problem since it requires only observations from the healthy samples. The rational idea behind OCSVM is to map the data into a high-dimensional feature space via a kernel function and then learn an optimal decision boundary that separates the training positive observations from the origin.

Given a set of training data $X={\left\{x_{i}\right\}}_{i=1}^{n}$, with *n* being the number of samples, OCSVM maps these samples into a high-dimensional feature space using a function ϕ through the kernel $K\left(x_{i},x_{j}\right)=\phi {\left(x_{i}\right)}^{T}\phi \left(x_{j}\right)$. Then, OCSVM learns a decision boundary that maximally separates the training samples from the origin. The primary objective of OCSVM is to optimize the following equation:

(6) $$\begin{array}{c} \max_{w,\xi ,\rho }-\frac{1}{2}{∥w∥}^{2}-\frac{1}{\nu n}\sum_{i=1}^{n}\xi_{i}+\rho , \end{array}$$

$$\begin{array}{c} s.t.\;w.\phi \left(x_{i}\right)\geq \rho -\xi_{i},\;\xi_{i}\geq 0,\;i=1,\ldots ,n, \end{array}$$

where ν (0<ν<1) is a user defined parameter to control the rate of anomalies in the training data, $\xi_{i}$ are the slack variable, $\phi (x_{i})$ is the kernel matrix and $w.\phi (x_{i})-\rho$ is the separating hyperplane in the feature space.

The problem turns into a dual objective by introducing Lagrange multipliers $\alpha =\{\alpha_{1},\cdots ,\alpha_{n}\}$. This dual optimization problem is solved using the following quadratic programming formula:

(7) $$\begin{array}{c} W=\min_{W(\alpha ,\rho )}\frac{1}{2}\sum_{i}^{n}\sum_{j}^{n}\alpha_{i}\alpha_{j}\phi \left(x_{i},x_{j}\right)+\rho \left(1-\sum_{i}^{n}\alpha_{i}\right), \end{array}$$

$$\begin{array}{c} s.t.\;0\leq \alpha_{i}\leq 1,\;\sum_{i=1}^{n}\alpha_{i}=\frac{1}{\nu n}, \end{array}$$

where $\phi (x_{i},x_{j})$ is the kernel matrix, α are the Lagrange multipliers and ρ is known as the bias term.

The partial derivative of the quadratic optimization problem (defined in Equation (7)) with respect to $\alpha_{i}$ is then used as a decision function to calculate the score for a new incoming sample:

(8) $$\begin{array}{c} g\left(x_{i}\right)=\frac{\partial w}{\partial \alpha_{i}}=\sum_{j}\alpha_{i}\phi \left(x_{i},x_{j}\right)-\rho . \end{array}$$

The OCSVM uses Equation (9) to identify whether a new incoming point belongs to the positive class when returning a positive value, and vice versa if it generates a negative value:

(9) $$\begin{array}{c} f\left(x_{i}\right)=\mathrm{sgn}\left(g\left(x_{i}\right)\right). \end{array}$$

### 3.4. Damage Detection and Severity Assessment

Given vibration data collected from multiple sensors when a structure is in a healthy case, tensor analysis is used to fuse and extract damage sensitive features from all these sensors. A OCSVM model is trained using a time matrix *C* decomposed from a healthy training tensor. When new data come in, which are associated with a new row in *C*, the new row will be estimated using the approach described in Section 3.1.3, and it will be fed to the trained model for damage detection. A negative decision value indicates that the structure behavior has changed (i.e., damage occurs) and vice versa.

For damage severity assessment, we analyze decision values returned from the OCSVM model. The rationality is that a structure with more severe damage (e.g., a longer crack) will behave differently from normal behaviour. Different ranges of the decision values may present different severity levels of damage.

For features using Wavelet Packet Energy, we also used OCSVM for damage detection and assessment in order to compare with the OCSVM model using tensor analysis.

## 4. Experimental Results

This section demonstrates how the combination of tensor-based features and OCSVM can successfully detect and assess the severity of structural damage. It is using the sensor-based data from the two case studies described in Section 2.

For all experiments, we have used the core consistency diagnostic technique (CORCONDIA) method described in [22] to decide the number of latent factors *R* in the CP method. This method suggested R=2 for all experimented datasets. The Gaussian kernel, defined in Equation (10), was employed in OCSVM since it has gained much more popularity in the area of machine learning and it has turned out to be an appropriate setting for OCSVM [23]. The Gaussian kernel parameter denoted by σ was set to the default value, and the ν parameter in Equation (6) was set to 0.01:

(10) $$\begin{array}{c} K\left(x_{i},x_{j}\right)=\exp\left(-\frac{{∥x_{i}-x_{j}∥}^{2}}{2\sigma^{2}}\right). \end{array}$$

The accuracy values were obtained using the F-Score (FS), defined as $F-\mathrm{score}=2\cdot \frac{\mathrm{Precision}\times \mathrm{Recall}}{\mathrm{Precision}+\mathrm{Recall}},$ where $\mathrm{Precision}=\frac{\mathrm{TP}}{\mathrm{TP}+\mathrm{FP}}$ and $\mathrm{Recall}=\frac{\mathrm{TP}}{\mathrm{TP}+\mathrm{FN}}$ (the number of true positive, false positive and false negative are abbreviated by TP, FP and FN, respectively).

### 4.1. The Cable-Stayed Bridge

Our tensor-based approach was validated using vibration data collected from the cable-stayed bridge described in Section 2. This case-study used 24 uni-axial accelerometers, which collected 262 samples (events). Each event consists of acceleration data for a period of 2 s at a sampling rate of 600 Hz. The magnitude of the uni-axial accelerometer data was normalized to have zero mean and unity variance before transforming the data into frequency domain using fast Fourier transform (FFT). The measured vibration responses for each sample resulted in a vector with 600 attributes representing the frequencies of each sample. The resultant three-way tensor data has a structure of 24×600×262.

The collected 262 samples were separated into two main groups, Healthy (125 samples) and Damaged (137 samples). The Damaged group was further partitioned into two different damaged cases: the “Car-Damage” emulated by the stationary car (107 samples) and the “Bus-Damage” emulated by the stationary bus (30 samples). Eighty percent of the healthy events (100 samples) from each sensor were randomly selected as a training tensor $X\in {ℜ}^{24\times 600\times 100}$ (i.e., *training* set). The samples related to the two damage cases (137) were added to the remaining 20% of the healthy data to form a *testing* set, which was then used for the model evaluation.

The ALS method described in Algorithm 1 was used to decompose the training tensor X into three matrices *A*, *B* and *C*. The matrix $C\in R^{100\times 2}$ represents data in time mode. These data were then used to construct an anomaly detection model using OCSVM. For each new incoming $X_{new}$ datum, we used Equation (3) to calculate $C_{new}$ that represents the tensor-based features. The decision function defined in Equation (9) is then used to generate a health score for $C_{new}$ and to specify whether this new event is healthy or damaged.

**Figure 6:**
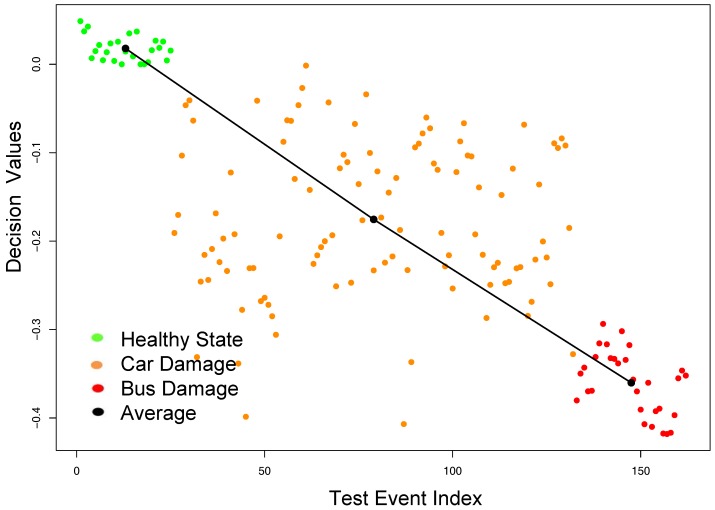
Damage identification results using tensor features on the cable-stayed bridge dataset.

Our constructed model using the tensor-based features was able to successfully detect all the healthy and damage events in the *testing* data set, and achieved an F-Score of 100%. Moreover, this model was able to assess the progress of the damage severity in the structure using the tensor-based features. To illustrate this, we calculated decision values for all test samples that were shown in Figure 6. The horizontal axis indicates the index of the test samples and the vertical axis indicates the magnitude of the decision value. A positive value indicates a sample classified as healthy, whereas a negative value indicates an event classified as damage.

The first 25 events, shown in green, refer to the healthy samples, i.e., before the presence of damage. The next 107 samples, shown in orange, refer to the car-emulated damaged samples. The following 30 samples, shown in red, refer to the bus-emulated damaged samples. The mean of all the decision values for each category was calculated and illustrated in Figure 6. A solid black line was constructed to connect the mean values. As can be seen from Figure 6, considering the effect of environmental and operational changes, the constructed OCSVM model using the tensor features was able not only to reliably separate the healthy state from a very slight damage case (“Car-Damage"), but also to assess the damage severity from “Car-Damage” to “Bus-Damage”. The decision values were further decreased for the samples related to the more severe “Bus-Damage”.

**Figure 7:**
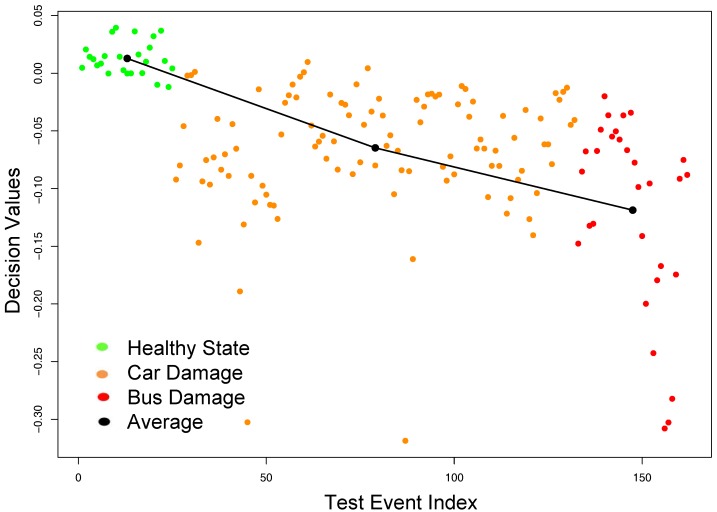
Damage identification results using WPE features on the cable-stayed Bridge dataset.

To illustrate the effectiveness of our tensor approach for data fusion and feature extraction, we compare the classification results of OCSVM using tensor-based features to the performance of OCSVM using WPE-based features, which was described in Section 3.2. The same *training* data set as above was used to extract the damage sensitive features using the WPE method and construct an OCSVM model. Similarly, the same previous *testing* data set was used to evaluate the classification performance of OCSVM using WPE features. The F-score accuracy of OCSVM was recorded at 97%. Moreover, the OCSVM decision values were not able to clearly assess the progress of the damage severity in the structure as illustrated in Figure 7.

### 4.2. The Jack Arch Specimen

Our second experiments were conducted using the vibration data acquired from six accelerometers instrumented on the specimen as described in Section 2. We applied our novel approach on this data set to evaluate the classification performance of OCSVM using the tensor-based features. The magnitude of the (x,y,z) from the tri-axial accelerometer reading was calculated and then normalized to have zero mean and unity variance. The Fourier transform method was then used to represent the data in frequency domain. The differences between vibrations of the three sensors in each node in the frequency domain were used as frequency variables. These variables yield better representation of the signal since the three accelerometers would move together if the structure is healthy and differently or independently otherwise. The collected data set comprised of 950 samples (a.k.a. events) separated into two main groups: Healthy (190 samples) and Damaged (760 samples). Each event consists of acceleration data for 3 s at 500 Hz, resulting in a vector of 750 frequency values. The damaged cases were partitioned into four different sub-cases of 190 samples, which each corresponds to a level of damage severity (i.e., 1 for the minimum damage and 4 for the maximum damage).

We randomly selected 80% of the healthy events (152 samples) from six sensors as a training tensor $X\in {ℜ}^{6\times 750\times 152}$ (i.e., *training* set). The remaining 20% of the healthy data and the data obtained from the four damage cases were used for testing (i.e., *testing* set). We applied the ALS method described in Algorithm 1 to decompose the tensor X into three matrices *A*, *B*, and *C*, which matrix *C* was used to construct an OCSVM model. For each arriving $X_{new}$ datum, we used Equation (3) to calculate $C_{new}$ that represents the tensor-based features. The decision function defined in Equation (9) was then used to generate a health score for $C_{new}$ and to specify whether this new event was healthy or damaged.

These experiments produce an F-score of 96% as a classification accuracy of the OCSVM model. Table 1 shows the resulting final confusion matrix from these experiments. The OCSVM model was able to detect 92.5% of the damage cases knowing that most of the missed 57 samples are related to damage case 1. It should be emphasized that the level of damage in this case study is considerably small with less than 0.5% reduction in the first natural frequency.

**Figure 8:**
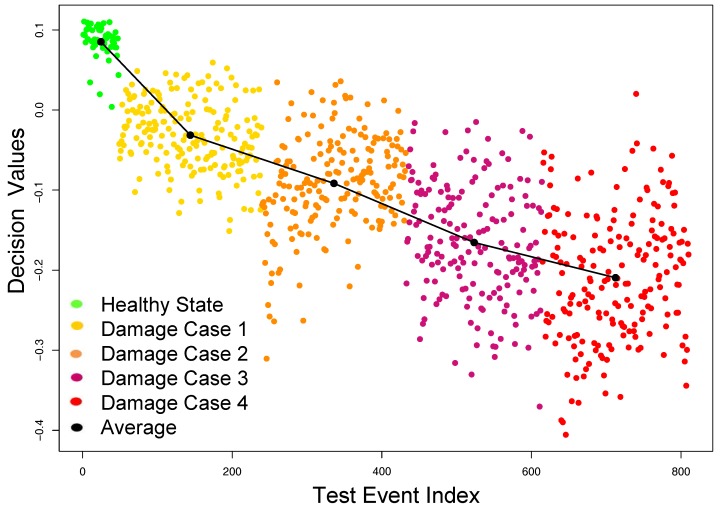
Damage identification results using tensor features on the specimen dataset.

In addition to the ability of identifying small defects, tensor-based features also have the capability to assess the progress of the damage severity in the structure based on the decision values obtained from OCSVM. It can be clearly observed from Figure 8 that the more severe the damage, the more negative the decision values (i.e., the data were more deviated from the training data). It is illustrated by a solid black line in Figure 8, which connects the means of all the decision values for each category.

**Figure 9:**
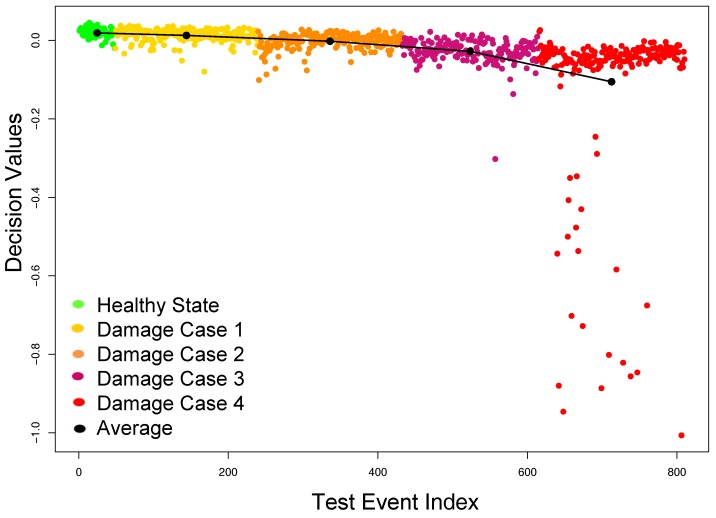
Damage identification results using WPE features on the specimen dataset.

The next experiment on this dataset was to compare the classification results of OCSVM using tensor-based features to the performance of OCSVM using WPE-based features. The same *training* and *testing* sets as above were used to extract the WPE-based feature and build the OCSVM model, and evaluate the performance of this alternative approach. The F-score accuracy of OCSVM was recorded at 76% and Table 2 shows the resulting confusion matrix for this experiment. The OCSVM model was only able to detect 61.1% of the damage cases. Further exploration of these results show that the WPE-based model missed 88.5% of the damage samples related to damage case 1 and 60% of the damage samples corresponding to damage case 2. Moreover, OCSVM decision values were not able to clearly assess the progress of the damage severity in the structure as illustrated in Figure 9.

## 5. Conclusions

This paper presented a novel method to analyse the data from a sensor-based SHM system in order to detect and assess damage in an infrastructure such as a bridge. Our contribution is three-fold.

First, we proposed a new algorithm that detects damage by using multi-dimensional data collected from distributed sensors on a structure. Our algorithm first applies tensor analysis to the acceleration data from different sensors, and combine them into a single feature vector. This feature is used as the input to build a OCSVM model. In the final step, our algorithm compares any new incoming data to this OCSVM model. If that new data point is oustide the model’s boundaries, then our method raises an anomaly event as the data most probably indicate the presence of a damage within the structure. In contrast to other recent SHM damage detection methods, our contribution is completely data-driven.

Second, we deployed an extensive SHM system on a cable-stayed bridge in operation in Western Sydney, and on a laboratory specimen, which replicates a substructure of the Sydney Harbour Bridge (SHB). The sensors and nodes on this latter case are similar to the ones we used in a previous deployment on the entire Sydney Harbour Bridge. We induced emulated and real damage in these two case studies, and collected large data sets with and without these damages. These data sets will be made available to the community.

Finally, we used these collected data to evaluate our approach and compare it against an alternative method, which uses a feature from the wavelet energy spectrum of the data. The results showed that our approach succeeded at detecting more damage events in both cases, with 100% vs. 97%, and 92.5% vs. 61.1%, respectively. Thus, our method outperformed a domain expert guided feature selection (e.g., wavelet energy spectrum) in both laboratory and real-world deployment cases.

We are exploring four different research directions as part of our future work. First, we would like to fully investigate the performance of the method for locating damage. We have obtained some initial success in this regard for some datasets; however, this work is still ongoing.

Second, we will deploy our novel method in our current SHM deployment on the SHB. This SHB deployment is a multi-tiered sensor network, i.e., sensor nodes are grouped into 10, and groups are connected to individual power unit devices, which are under two gateway devices linking to a cloud server. Thus, one challenge is to find the optimal level to deploy the tensor building step, i.e., on a leader node within a group, or on a gateway, etc. Third, we will also investigate the application of our method to other structures, such as building or road segments. We have ongoing collaboration with other academic and industry entities, which will provide us with the building and road data for this future work. We will finally explore the application of our algorithm to other types of sensor collected data, such as strain gauge or acoustic vibration.

## Figures and Tables

**Table 1:** Resultant confusion matrix of OCSVM using tensor-based features on the specimen dataset.

	Damage	Healthy
Damage	703	0
Healthy	57	48

**Table 2:** Resultant confusion matrix of OCSVM using WPE-based features on the specimen dataset.

	Damage	Healthy
Damage	465	4
Healthy	295	44
